# Neuronal Nitric Oxide Synthase Is Dislocated in Type I Fibers of Myalgic Muscle but Can Recover with Physical Exercise Training

**DOI:** 10.1155/2015/265278

**Published:** 2015-03-17

**Authors:** L. Jensen, L. L. Andersen, H. D. Schrøder, U. Frandsen, G. Sjøgaard

**Affiliations:** ^1^Institute of Sports Science and Clinical Biomechanics, University of Southern Denmark, 5000 Odense C, Denmark; ^2^Institute of Clinical Research, Pathology and SDU Muscle Research Cluster, University of Southern Denmark, 5230 Odense M, Denmark; ^3^National Research Centre for the Working Environment, 2100 Copenhagen, Denmark

## Abstract

Trapezius myalgia is the most common type of chronic neck pain. While physical exercise reduces pain and improves muscle function, the underlying mechanisms remain unclear. Nitric oxide (NO) signaling is important in modulating cellular function, and a dysfunctional neuronal NO synthase (nNOS) may contribute to an ineffective muscle function. This study investigated nNOS expression and localization in chronically painful muscle. Forty-one women clinically diagnosed with trapezius myalgia (MYA) and 18 healthy controls (CON) were included in the case-control study. Subsequently, MYA were randomly assigned to either 10 weeks of specific strength training (SST, *n* = 18), general fitness training (GFT, *n* = 15), or health information (REF, *n* = 8). Distribution of fiber type, cross-sectional area, and sarcolemmal nNOS expression did not differ between MYA and CON. However, MYA showed increased sarcoplasmic nNOS localization (18.8 ± 12 versus 12.8 ± 8%, *P* = 0.049) compared with CON. SST resulted in a decrease of sarcoplasm-localized nNOS following training (before 18.1 ± 12 versus after 12.0 ± 12%; *P* = 0,027). We demonstrate that myalgic muscle displays altered nNOS localization and that 10 weeks of strength training normalize these disruptions, which supports previous findings of impaired muscle oxygenation during work tasks and reduced pain following exercise.

## 1. Introduction

Musculoskeletal disorders are among the most frequent and costly health care problems in North America and Europe [1]. Work-related neck/shoulder pain, particularly, chronic pain, tightness, and tenderness of the trapezius muscle, trapezius myalgia, is common in female employees performing repetitive work tasks [2, 3]. Women with trapezius myalgia show increased muscle weakness [4, 5] and muscle fatigue [6]. Trapezius myalgia is associated with various pathological changes including mitochondrial changes in type I fibers [7, 8], ragged-red-fibers [9], altered satellite cell distribution [10], and reduced capillarization per fiber area [7, 8, 11]. Furthermore, numerous studies have shown that trapezius myalgia is accompanied by poor oxygenation, focal cell death, and disrupted metabolic homeostasis [8]. Taken together these data have led to the development of the “Cinderella theorem,” which proposes that selected type I fibers are the first to be recruited during repetitive movements at low static tension and, consequently, these motor units are constantly activated leading to overload of single muscle fibers [2]. The theory is supported by the finding of an increased proportion of hypertrophied type I mega fibers with poor capillarization [12] and a reduced capillary to fiber area [3] in trapezius myalgia. Overall, these intramuscular changes create disturbances in energy homeostasis and cellular hypoperfusion of enlarged type I fibers has been suggested as a major factor in the development of muscle pain [13, 14].

Nitric oxide (NO) is a transient multipurpose signalling molecule synthesized from oxygen and L-arginine by the muscle specific neuronal nitric oxide synthase (nNOS) [15–18]. In* m. vastus lateralis*, nNOS is anchored at the sarcolemma by binding of a PDZ domain to *α*1-syntrophin, a member of the dystrophin-glycoprotein complex (DGC), and interacting with dystrophin [19, 20]. NO is involved in regulating satellite cell activation, muscle development, metabolism, muscle contraction, and blood flow [21]. Recent studies indicate that nNOS together with endothelial NOS (eNOS) is responsible for regulation of microvascular tone in the muscle [22, 23] and nNOS synthase activity is tightly controlled by calmodulin and efflux of intercellular Ca^++^ [24]. Copp and colleagues demonstrated the importance of nNOS-derived NO during contractions in healthy rat spinotrapezius muscle using the selective nNOS inhibitor SMTC [25] and the role of the bioavailability of NO during recovery from skeletal muscle contractions using NO donors/inhibitors [26], the latter supporting a mechanistic link between a reduced NO availability and prolonged muscle metabolic recovery.

Changes in expression and location of nNOS have been associated with development of pathogenesis and disease progression in a number of myopathies and membrane-associated gene defects [27, 28]. Muscular dystrophies arising from sarcolemmal defects, like Duchenne muscular dystrophy (DMD) and limb-girdle muscular dystrophy (2C, 2D, and 2E), where the DGC is detached from the sarcolemma, show loss of nNOS from the sarcolemma [29–31]. More surprisingly, other forms of non-DGC-related muscular dystrophies, myopathies, and neuromuscular disorders also display mislocalization of sarcolemmal nNOS [32, 33]. These diseases vary in severity but are generally characterized by progressive muscle loss, increasing weakness, and muscle fatigue.

Previous data propose that loss of nNOS in DMD give rise to a dysregulation of vasoconstriction/blood flow [34]. In line with this, Finanger and colleagues suggest that loss of sarcolemmal nNOS is related to both impaired mobility and catabolic stress and propose that sarcolemmal nNOS may be significant in maintaining muscle homeostasis [33]. Studies on the muscle specific nNOS knockout mouse show that these mice have a lower maximum isometric force and show increased fatigability [35], and loss of both dystrophin and nNOS causes impaired control of blood flow during exercise [36]. In mice, nNOS/NO mediates muscle atrophy via regulation of FOXO transcription factors and dislocation of nNOS to the sarcoplasm after disuse-induced studies in the nNOS-null mice [37]. However, no studies have investigated the role of these mechanisms in trapezius myalgia.

In healthy muscle, production of NO plays a role in inhibition of sympathetic vasoconstriction under normal physiological conditions [38]. As a result of loss of nNOS the normal protective action of NO against local ischemia during contraction induced calcium concentrations is ambulated and an increase in cellular susceptibility to metabolic stress may be the consequence [34, 39].

The underlying causes of trapezius myalgia and development of chronically painful muscles remain unclear, but multiple studies have demonstrated that strengthening exercises for the neck and shoulders reduce the associated pain [40–42]. Accordingly, the aim of the present study was to investigate pathological changes in trapezius myalgia that can help explain the underlying mechanisms of chronic muscle pain and to reveal possible actions to alleviate such pain. Specifically, the following hypotheses were tested for women clinically diagnosed with trapezius myalgia (MYA) compared with healthy controls (CON): (1) MYA patients have decreased sarcolemma-localized nNOS in type I fibers compared to CON; (2) MYA patients have increased sarcoplasm-localized nNOS in type I fibers compared to CON; and (3) strength training normalizes nNOS expression in MYA toward the level in CON.

## 2. Materials and Methods

### 2.1. Study Design and Participants

In the case-control study 42 women clinically diagnosed with trapezius myalgia (MYA patients: age 44 ± 8 yrs, height 165 ± 6 cm, weight 72 ± 15 kg, and days with neck pain during previous year 219 ± 19 days) and 18 healthy controls (CON: age 45 ± 9 yrs, height 167 ± 6 cm, weight 70 ± 11 kg, and days with neck pain during previous year 5 ± 6 days) were included. One subject was omitted from the study prior to analysis due to insufficient biopsy material resulting in 41 subjects in MYA. All participants were active in the labour market and recruited from workplaces with monotonous and repetitive work tasks. Exclusion criteria were previous trauma, life threatening diseases, whiplash injury, cardiovascular diseases, or arthritis in the neck and shoulder. All participants went through a clinical investigation of the neck and shoulder, performed by trained clinical personnel, who worked together as a calibrated team as described in [43]. The main criteria for a clinical diagnosis of trapezius myalgia were (1) chronic or frequent pain in the neck area, (2) tightness of the upper trapezius muscle, and (3) palpable tenderness of the upper trapezius muscle. The CON group showed no signs of pain in the neck area, tightness of the upper trapezius muscle, or palpable tenderness of the upper trapezius muscle.

In the randomized controlled trial (RCT) forty-eight women with trapezius myalgia were included and randomized to one of three 10-week intervention groups: 14 to a reference group (REF), 18 to specific strength training (SST), and 16 to general fitness training (GFT). Unfortunately, six participants dropped out of the REF group after allocation and inadequate biopsy material was available at baseline for one subject in REF and for one subject in the GFT and at followup for 2 subjects in each of the groups GFT and SST. This resulted in biopsies from 36 women in the study being analysed in a paired statistical design: *n* = 7 for REF, *n* = 16 for SST, and *n* = 13 for GFT. Further details on the study group have previously been reported [42].

All participants were informed about the purpose of the study and gave written informed consent before their participation. The study was conducted according to the Declaration of Helsinki and was approved by the local ethics committee of Copenhagen, Denmark (KF 01-138/04). Furthermore the study was registered in the International Standard Randomised Controlled Trial Number Register: ISRCTN87055459.

### 2.2. Intervention Protocol

The participants in the RCT were divided into three groups as described in detail [42]. One group (SST, *n* = 18) performed high-intensity specific strength training with five dumbbell exercises for the shoulder and neck muscles (one-arm row, shoulder abduction, shoulder elevation, reverse flyes, and upright row) for 20 min three times a week. During each session three of the five different exercises were performed for three sets of each exercise with relative loadings of 8–12 repetitions maximum in a periodized and progressive manner. The specificity and high level of muscle activation of these exercises have been documented previously [44]. A second group (GFT, *n* = 16) performed general fitness training on a leg-bicycle ergometer with relative loadings of 50–70% of the maximal oxygen uptake for 20 min three times a week. The cycling was performed in an upright position with relaxed shoulders. A third group (REF, *n* = 8) was not offered any physical training but received information about health-promoting activities for a total of 1 h per week.

### 2.3. Functional Description of Subjects

Results from the case-control and RCT study on individual pain perception and muscle function have previously been published. For comparison with the data presented here it should be underlined that MYA demonstrated in the neck/shoulder area a higher pain level [45] and lower muscle strength as well as lower muscle activation based on EMG measures [4, 5] compared with CON. In the RCT study SST reduced pain (~80%, *P* < 0.001) [42], improved muscle strength and activation (peak torque increased by 18–29%, *P* < 0.01; rate of torque development increased by 61–115%, *P* < 0.001) [46], improved muscle endurance, and increased capillarization [6].

### 2.4. Sample Collection

Muscle biopsies from MYA and CON were collected under local anaesthesia (1% lidocaine) with a Bergstrom biopsy needle from the upper* trapezius* muscle at the midpoint between the 7th cervical vertebra and the acromion. The exact biopsy site was determined by inspection with ultrasonography. Approximately 50 mg tissue was excised, quickly dissected from fat and connective tissue; fibres were aligned, embedded in Tissue-Tek (Sakura Finetek Europe, Zoeterwoude, Netherlands), and frozen in precooled isopentane. Samples were stored at –80°C. One muscle biopsy collected from the* vastus lateralis* of a healthy control subject was treated and stored as described above and was used as comparison for the nNOS staining. All biopsy samples were assigned a unique identification number, thus blinding the investigator to the participant's identity. Transverse sections (10 *μ*m) were cut at –24°C using a cryostat and picked up onto SuperFrost Plus glass slides (Menzel-Gläser, Braunschweig, Germany). Biopsies were stained in batches, arranged by an investigator not involved in the analysis, such that each batch contained biopsies from all 3 groups and the samples before and after training from the same individuals.

### 2.5. Histochemical Staining

*Diaphorase Activity*. nNOS activity was evaluated by nicotinamide adenine dinucleotide phosphate-tetrazolium reductase staining (NADPH-TR) as described previously [20]. Sections of frozen tissue were fixed in freshly prepared 4% paraformaldehyde before they were incubated with NADPH-TR solution (nitroblue tetrazolium 0.2 mM (Sigma) and *β*-NADPH 1 mM (Sigma) in Tris buffer 0.2 M with 0.25% triton X-100, pH 7.3, sterile filtered) for 60 min at 37°C, washed in Tris buffer 0.02 M pH 7.4, and mounted in aqueous mounting medium (Vector Laboratories, VWR, Herlev, Denmark).

*NADH Oxidase Activity*. Nicotinamide adenine dinucleotide-tetrazolium reductase (NADH-TR) stains were performed according to standard procedures. Briefly, frozen sections were incubated with NADH-TR solution (nitroblue tetrazolium 2 mM (Sigma) and NADH 1 mM (Sigma) in Tris buffer 0.02 M pH 7.4, sterile filtered) for 45 min at 37°C and washed in Tris buffer 0.02 M pH 7.4 and H_2_O followed by 10 min incubation with calcium chloride in 4% formaldehyde. The sections were washed in H_2_O and fixed in acetone before aqueous mounting.

### 2.6. Immunohistochemical Staining

Sections of frozen tissue were fixed in 4% normal buffered formalin and blocked in Protein Block (Dako, Denmark) before they were incubated with primary antibodies 1 h at RT. For the nNOS/MHC-II staining, the same batches of primary and secondary antibodies were used for all sections. Primary antibodies for myosin heavy chain II (MHC-II) (1 : 2000, M4276, Sigma Aldrich, Denmark), nNOS (1 : 200, AF2416, R&D Systems, United Kingdom), myosin heavy chain I (MHC-I) (1 : 2000, M8421, Sigma Aldrich, Denmark), *α*-dystroglycan (1 : 50, clone VIA4-1, Millipore, Germany), dystrophin (rod, 1 : 10, MAB1692, Millipore, Germany; C-terminal, 1 : 5, MAB1694, Millipore, Germany; N-terminal, 1 : 20, clone 34C5, Novocastra, United Kingdom), or laminin (polyclonal, Z0097, Dako, Denmark) were used. Alexa Fluor 488 or 555 donkey anti-mouse (1 : 1000, Molecular Probes; Invitrogen A/S, Denmark), 555 rabbit anti-goat (1 : 1000, Molecular Probes; Invitrogen A/S, Denmark), or 350 donkey anti-rabbit (1 : 500, Molecular Probes; Invitrogen A/S, Denmark) were used as secondary antibodies, while 4′,6-diamidino-2-phenylindole (DAPI) in the mounting medium (Molecular Probes ProLong Gold antifade reagent, Invitrogen) was used to stain the nuclei blue. The fiber specificity of MHC-I and MHC-II antibodies was evaluated to ensure specificity to type I and type II fibers, respectively. Images can be found in Supplementary Material (Figure  S1) (see Supplementary Materials available online at http://dx.doi.org/10.1155/2015/265278).

### 2.7. Image Collection and Data Analysis

Randomly, three distinct images were collected from each section stained with MHC-II/nNOS using Axio Imager 1 equipped with AxioVision 4.6 software at identical exposure settings allowing a direct comparison between sections. For each image the cross-sectional area (CSA) and total number of fibres were determined. The relative number of type II fibres on each section was quantified by counting the number of type II^+^ fibres and expressing this number relative to the total number of muscle fibres. Additionally, semiquantitative analysis of sarcolemma-localized nNOS intensity was performed by grouping each fibre into three categories after visual inspection and describing the fibres as no or very little nNOS expression (nNOS^−^), reduced expression (nNOS^+^), or normal expression (nNOS^++^) and likewise expressing each group relative to the total number of muscle fibres. For each subject, the CSA of the fibres belonging to nNOS^−^ was determined (CSA^(nNOS−)^) and the total number of fibres showing nNOS staining in the sarcoplasm was quantified (nNOS^sp^) and expressed relative to the total number of muscle fibres. For the remaining staining representative images were collected.

### 2.8. Validation and Statistics

The investigator collecting the biopsy images and performing the analyses was blinded to experimental setup and a fraction of the biopsies were analysed twice to ensure consistency in counting and analysis. Subsequently, the data were unblinded and statistically analysed using paired (RCT) or unpaired (case-control)* t*-test in GraphPad Prism 5. Statistical significance was accepted at an alpha level of 0.05. Data are presented as mean ± standard deviations (SD) unless stated otherwise.

## 3. Results

**Figure 1:**
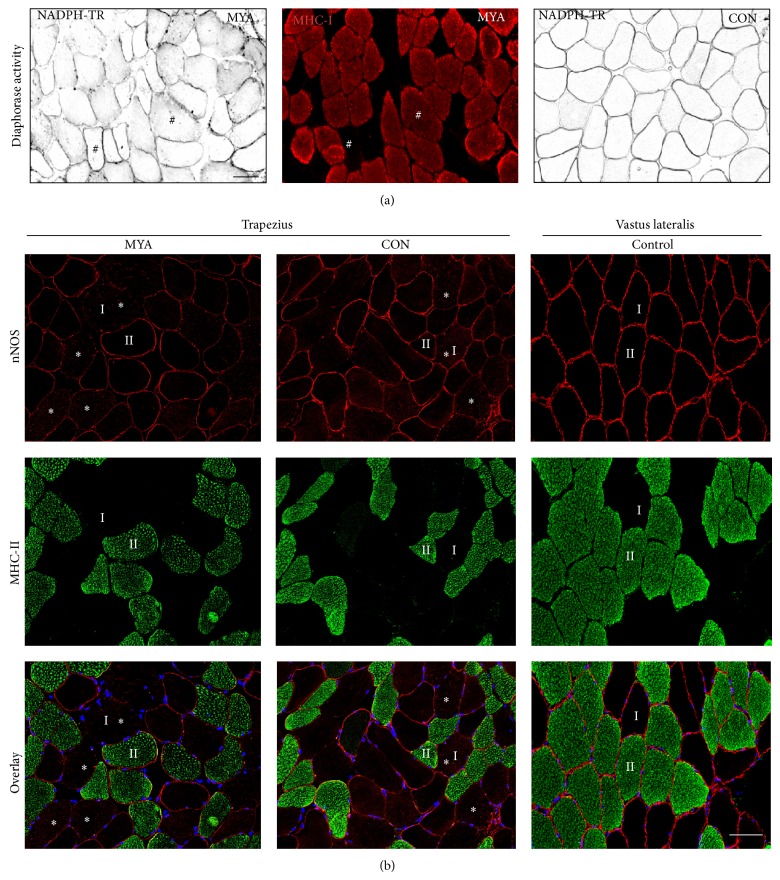
Altered nNOS activity and protein expression. (a) Assessment of NOS activity in trapezius muscle from women with repetitive work tasks (MYA patients and CON) shows loss of activity in sarcolemma and gain of activity in sarcoplasm of large type I fibers compared to type II. Serial sections show NOS activity in black and MHC-I in red. # marks identical fibers on serial sections. Scale bar: 50 *μ*m. (b) Immunohistochemical staining of nNOS and MHC-II from trapezius and vastus lateralis muscle. In trapezius muscle type I fibers show reduced sarcolemma-localized nNOS protein and greater sarcoplasmic nNOS protein (∗) compared with type II fibers. Vastus lateralis did not show this type of alterations. nNOS in red, myosin heavy chain II in green, and a merged image with DAPI in blue. I and II mark identical type I and type II fibers, respectively. Scale bar: 50 *μ*m.

**Figure 2:**
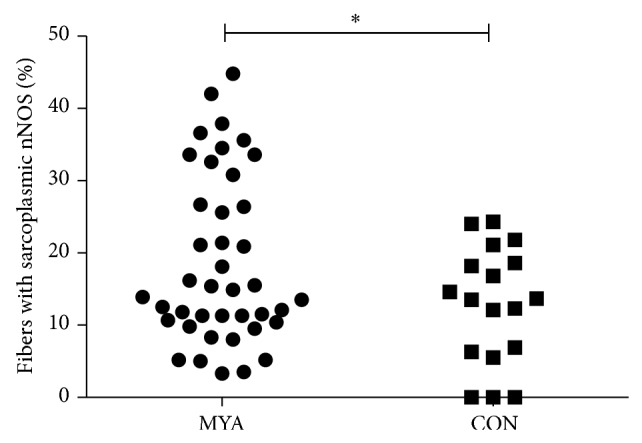
Trapezius myalgia shows sarcoplasm-localized nNOS. Data from the case-control study showed an increased proportion of muscle fibers with nNOS protein localized in the sarcoplasm in trapezius myalgia (MYA) patients compared to healthy controls (CON) detected by immunohistochemistry. 18.8 ± 12 versus 12.8 ± 8%, *P* = 0.049.

The number of fibers counted per subject was 292+/− 43 (MYA) and 302+/− 82 (CON). No difference was observed in distribution of type I/II fibers and mean CSA between MYA patients and CON (Table 1). NADPH diaphorase activity staining of trapezius muscle revealed lower nNOS activity in the sarcolemma and higher activity in the sarcoplasm of type I fibers compared to type II fibers (Figure 1(a)). Based on evaluations of immunohistochemical staining of nNOS from all subjects, approximately 50% of the muscle fibers in both MYA and CON showed either loss of (nNOS^−^) or reduced amounts of (nNOS^+^) sarcolemmal-localized nNOS (Table 1; Figure 1(b)). The remaining 50% of muscle fibers expressed normal levels of nNOS (nNOS^++^). Notably, an increased proportion of fibers in MYA presented with nNOS protein localized to the sarcoplasm compared to CON (18.8 ± 12 versus 12.8 ± 8%, *P* = 0.049) (Table 1 and Figure 2). For purposes of comparison with trapezius muscle an image of nNOS expression in healthy vastus lateralis muscle is included (Figure 1(b)).

**Figure 3:**
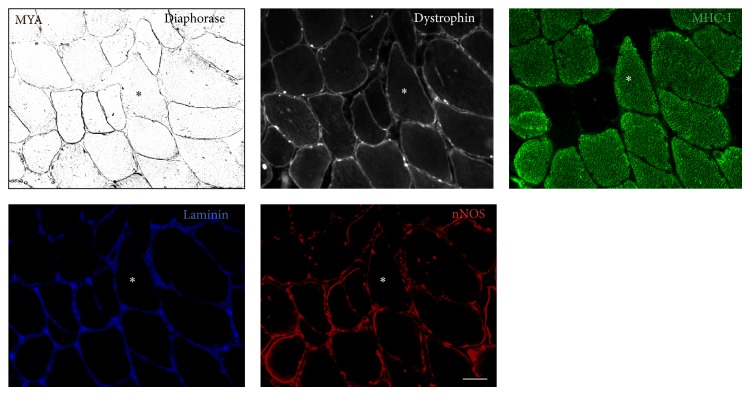
Intact dystrophin protein of type I fibers. Serial sections reveal normal dystrophin and laminin sarcolemmal protein expression in type I fibers displaying loss of NOS activity and reduced nNOS protein. NOS activity in black, dystrophin in white, laminin in blue, MHC-I in green, and nNOS in red. ∗ marks identical fibers on serial sections. Scale bar: 100 *μ*m.

Histochemical and immunohistochemical staining on serial sections for NADPH diaphorase activity, dystrophin (rod domain), MHC-I, laminin, and nNOS revealed normal dystrophin expression in type I fibers with loss of nNOS activity and loss of sarcolemmal nNOS protein (Figure 3). Staining for *α*-dystroglycan and N- and C-terminal domains of dystrophin revealed a similar pattern (images not shown).

**Figure 4:**
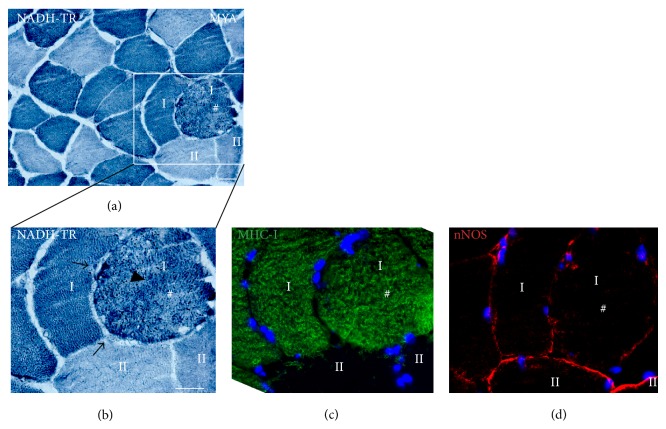
Intermyofibrillar network changes in muscle fibers lacking sarcolemmal nNOS. Enlarged type I fiber (#) with decreased sarcolemmal nNOS protein shows alterations in the intermyofibrillar network demonstrated by irregular NADH-TR staining ((a) and (b): arrow head). Note also a subsarcolemmal accumulation of cellular material or mitochondria seen as dark areas below the fiber membrane ((b): dark arrow). Type II fibers appear normal. NADH-TR (mitochondria) is blue, MHC-I is green, and nNOS is red. # marks identical fibers on serial sections. Scale bar: 50 *μ*m.

NADH-TR staining showed alterations in the intermyofibrillar network and clustering of cellular material in the sarcoplasm of some type I muscle fibers. Most of these same fibers showed loss of sarcolemmal nNOS (Figure 4).

Although the distribution of fibers belonging to nNOS^−^, nNOS^+^, or nNOS^++^ was not found to be different between MYA and CON (Table 1), it was observed that all fibers in nNOS^−^ were of type I (Figure 1). Moreover, the CSA of fibers in nNOS^−^ (CSA^nNOS−^) was significantly larger compared to the average CSA (CSA^average^) in both MYA and CON (CON: 5166 ± 1026 versus 5950 ± 1309 *μ*m^2^; MYA: 5149 ± 997 versus 6104 ± 1415 *μ*m^2^, *P* < 0.01, Table 1 and Figure 1).

**Figure 5:**
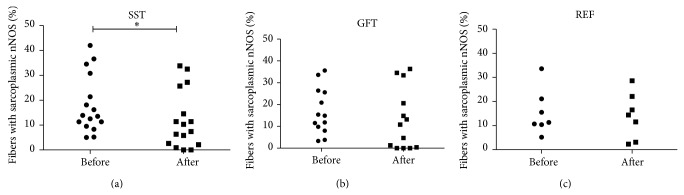
Effect of exercise on sarcoplasm-localized nNOS. The randomized controlled trial revealed that specific strength training (SST) caused a decrease in sarcoplasm-localized nNOS protein after 10 weeks of training ((a): before 18.1 ± 12 versus after 12.0 ± 12%; *P* = 0,027), while the group doing general fitness training (GFT) ((b): before 17.0 ± 11 versus after 13.1 ± 14%; *P* = 0.27) and the reference group (REF) ((c): before 15.4 ± 9 versus after 14.1 ± 10%; *P* = 0.70) did not change.

The randomized controlled trial study showed that 10 weeks of muscle training altered the nNOS expression. The group performing SST showed a significant decrease in sarcoplasm-localized nNOS after 10 weeks of training (before 18.1 ± 12 versus after 12.0 ± 12%; *P* = 0,027), while GFT (before 17.0 ± 11 versus after 13.1 ± 14%; *P* = 0.27) and REF (before 15.4 ± 9 versus after 14.1 ± 10%; *P* = 0.70) did not change (Figure 5).

## 4. Discussion

The major finding of this study is a decreased sarcolemmal nNOS expression of distinctive type I fibers in the trapezius muscle of women with repetitive work tasks. Further, women suffering from trapezius myalgia show more frequent localization of nNOS to the sarcoplasm compared to healthy controls and this sarcoplasmic localization can be reversed by specific strengthening exercises.

Trapezius muscle in women with repetitive work tasks (both with and without neck pain) displays a distinct loss of sarcolemma-localized nNOS in approximately one quarter of the muscle fibers (Table 1), which is unlike the nNOS pattern seen in healthy biopsies collected from m. vastus lateralis, where all fibers show clear and similar staining [47, 48]. Loss or disruption of sarcolemmal nNOS expression has earlier been detected in patients with various neuromuscular diseases and is in general correlated with disease progression [27–30, 33, 49, 50]. Previous data propose that loss of sarcolemmal nNOS in DMD leads to a dysregulation of vasoconstriction [34, 35] or is related to impaired mobility and catabolic stress and that nNOS is necessary for maintaining muscle homeostasis [33, 35]. It has been shown that alpha-adrenergic vasoconstriction was greatly impaired in the contracting muscles of the *α*-syntrophin null mice [51] and that reduced sarcolemmal nNOS exacerbates the fatigue experienced after mild exercise because the normal contraction induced decrease in local vasoconstriction is disrupted [32]. Further, nNOS has been shown to regulate basal microvascular tone in humans [52] and knockout of muscle specific nNOS in mice caused reductions in maximum tetanic force and increased susceptibility to contraction induced fatigue [35]. Together, these results support a role of nNOS in local vasoconstriction and development of functional ischemia and metabolic stress in the trapezius muscle during contraction and disruption of nNOS expression might subsequently play a role in development of work-related chronic muscle damage.

Previous analyses of fibers from the present subjects revealed the presence of type I mega fibers in trapezius muscle [12], defined as type I fibers being at least twice the size of the median type I muscle fiber size for each individual. In the present study, it is demonstrated that sarcolemma-localized nNOS is missing in enlarged muscle fibers. Moth-eaten fibers, which are evidence of intermyofibrillar network alterations, that is, changes in sarcoplasmic reticulum and mitochondria localization, have previously been found to have an increased CSA in the trapezius muscle [53]. In support of this finding, we observe that a great fraction of the moth-eaten fibers show disruption of sarcolemmal nNOS expression, thus implying that interrupted mitochondrial function and impaired local NO production and regulation occur specifically in enlarged type I fibers in the trapezius muscle of women with repetitive work tasks. Whether nNOS dislocation in large type I fibers is a result of muscle fiber hypertrophy caused by prolonged low force static work leading to functional ischemia and focal death or myofibrillar network changes and loss of sarcolemmal nNOS causing ischemia during low force static work and eventually inducing hypertrophy remains unknown.

In the present study, more frequently dislocation of nNOS to the sarcoplasm is observed in MYA patients compared to CON. This is contrary to a previous study, which found no difference in nNOS expression using whole muscle homogenate and western blotting analysis comparing fibromyalgia and controls [54]. The discrepancy may arise from difference in pathophysiology of the diseases and is further explained by increased ability and thus sensitivity to identify nNOS in specific locations using histochemistry and immunohistochemistry methods of detection as used in the present study. Previous studies imply that dislocation of nNOS and the following disruption of local NO production increase oxidative stress, glycosylate and/or nitrosylate the skeletal muscle ryanodine receptor (RYR1), and alter sarcoplasmic calcium release, which induces protein degradation and activation of calpains. Increased stress-related mRNA and protein [55] and decreased oxygenation [56] have been identified in the present study cohort and implicate functional defects. Dislocation of nNOS from the sarcolemma will leave unanchored nNOS floating around inside the cell, which is in agreement with our observations of sarcoplasmic nNOS. This sarcoplasmic localization is thought to increase the presence of reactive oxygen and nitrogen species (ROS/RNS) [47], and the present observation of alterations in intermyofibrillar network and a subsarcolemmal increase and/or clustering of mitochondria along the fiber membrane of type I fibers further supports this notion as mitochondria are a major source of ROS [57]. In combination, the increased size of type I fibers and thus increased diffusion distance leading to functional ischemia and the increased ROS/RNS might lead to modifications of proteins and overall tissue damage. Mutations in RYR1 gene leading to a defective RYR1 have been shown to cause neuromuscular disease and studies have shown an association between mutations in RYR1 causing disruptions in sarcoplasmic calcium regulation and development of unexplained rhabdomyolysis and/or exertional myalgia [58–60]. Further, several experiments indicate a relationship between muscle weakness and nitrosylation of RYR1 in muscular dystrophies [50, 61] and in the heart [62] stressing the importance of a tightly controlled NO production to maintain calcium homeostasis and correct muscle function. We found a normal expression and localization of dystrophin indicating maintained membrane integrity and ruling out disrupted membrane anchoring of dystrophin as the reason for dislocation of nNOS as seen in DMD.

Some of the early studies on trapezius myalgia found a link between a reduction in blow flow and an increase in muscle pain [63] and this observation has been supported and extended by others since then. Women with myalgia have lower muscle oxygenation, higher muscular lactate, and lower anaerobic threshold compared to controls [3, 7, 9, 11, 14, 53, 56, 64]. Multiple studies have found exercise training to reduce work-related pain in the neck and shoulder [65–68], including subjects from the present study cohort. The present data show a significant reduction in sarcoplasmic nNOS after SST. Further, SST also induced an increased capillarization, which likely also plays a role to increase blood flow and decrease pain [6, 12]. Previously, an association between nNOS and impaired mobility status has been detected in neuromuscular conditions [33], but the specific function of nNOS in exercising trapezius myalgia has not been investigated. A role for NO in exercise intolerance and impaired microcirculation has been suggested [25, 26, 69], and we have added to this theory based on results from the present study population. Together, they highlight the need for additional investigations particularly regarding the role of nNOS localization and local NO production in work-related muscle pain.

## 5. Conclusion

We showed that sarcolemmal nNOS expression is irregular and absent from selected fibers in the trapezius muscle. Moreover, we found an increase in sarcoplasm-localized nNOS in women with trapezius myalgia, which was essentially normalized by 10 weeks of specific strength training. Abnormalities in nNOS expression show a potential of predicting the progression of muscle damage and pain, and correcting the dislocation of nNOS may prove essential in treatment of work-related muscle pain as well as other muscle diseases.

## Supplementary Material

Figure S1: Specificity of MHC-I and MHC-II antibodies. Double staining of MHC-I and MHCII antibodies show no cross reactivity or double staining of fibers, indicating that the MHC-I antibody mark type I fibers exclusively, while MHC-II is specific for type II fibers. (A) Image from trapezius muscle. (B) Image from vastus lateralis muscle. Scalebar 100 *μ*m.

## Figures and Tables

**Table 1:** Quantification of immunohistochemical stainings.

	Fiber type percentage (%)		nNOS intensity (%)				Cross-sectional area (*µ*m^2^)	
	Type I	Type II	nNOS^−^	nNOS^+^	nNOS^++^	nNOS^sp^	Average	nNOS^−^ fibers
CON *n* = 18	63.3 ± 11	36.7 ± 11	25.3 ± 7	24.4 ± 8	50.2 ± 8	12.8 ± 8	5166 ± 1026	5950 ± 1309^b^
MYA *n* = 41	65.8 ± 11	34.2 ± 11	26.4 ± 10	27.2 ± 14	46.4 ± 12	18.8 ± 12^a^	5149 ± 997	6104 ± 1415^b^
REF *n* = 7								
Before	63.0 ± 10	37.0 ± 10	20.1 ± 7	39.4 ± 25	40.5 ± 18	15.4 ± 9	5362 ± 629	5995 ± 505^b^
After	64.1 ± 15	35.9 ± 15	28.6 ± 8	30.0 ± 17	41.3 ± 14	14.1 ± 10	5221 ± 989	6783 ± 1556^b^
GFT *n* = 13								
Before	67.2 ± 11	32.8 ± 11	31.5 ± 11	26.6 ± 11	41.9 ± 13	17.0 ± 11	5362 ± 525	6215 ± 1776^b^
After	65.6 ± 13	34.4 ± 13	30.8 ± 12	26.9 ± 11	42.9 ± 13	13.1 ± 14	5249 ± 1935	6106 ± 1437^b^
SST *n* = 16								
Before	66.0 ± 12	34.0 ± 12	22.4 ± 7	24.8 ± 7	52.9 ± 7	18.1 ± 12	4956 ± 1417	6211 ± 1489^b^
After	64.3 ± 13	35.7 ± 12	23.7 ± 9	25.9 ± 9	50.4 ± 9	12.0 ± 12^c^	5300 ± 1805	6123 ± 1192^b^

^a^Significant increase in nNOS^sp^ in MYA compared to CON (*P* < 0.05). ^b^Significant increase in CSA^nNOS−^ compared to CSA^avarage^ (*P* < 0.01). ^c^Significant decrease from before to after training (*P* < 0.05).

No nNOS in the sarcolemma (nNOS^−^), reduced nNOS in the sarcolemma (nNOS^+^), normal nNOS in the sarcolemma (nNOS^++^), and nNOS in the sarcoplasm (nNOS^sp^).

## Abbreviations

NO: Nitric oxide
nNOS: Neuronal nitric oxide synthase
MYA: Patients diagnosed with trapezius myalgia
CON: Control
GFT: General fitness training
SST: Specific strength training
REF: Health information
CSA: Cross-sectional area
DMD: Duchenne muscular dystrophy
DCG: Dystrophin-glycoprotein complex.
