# Supplementary figures and images for: Neuronal Nitric Oxide Synthase Is Dislocated in Type I Fibers of Myalgic Muscle but Can Recover with Physical Exercise Training
Source: Biomed Res Int. 2015 Mar 17;2015:265278. doi: 10.1155/2015/265278 (PMC4380094; doi:10.1155/2015/265278)

Figure S1

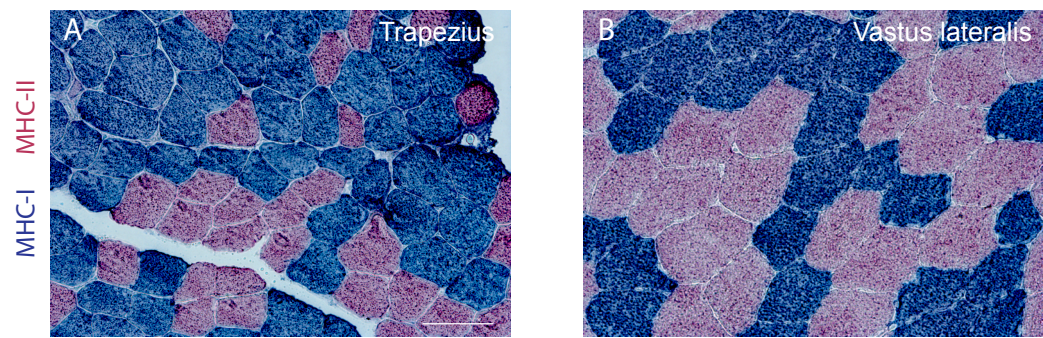

Supplement: Supplementary file 1 — Figure S1: Specificity of MHC-I and MHC-II antibodies. Double staining of MHC-I and MHCII antibodies show no cross reactivity or double staining of fibers, indicating that the MHC-I antibody mark type I fibers exclusively, while MHC-II is specific for type II fibers. (A) Image from trapezius muscle. (B) Image from vastus lateralis muscle. Scalebar 100 μm. [file 265278.f1.pdf]
